# A Smartphone App for Individual Xylazine/Ketamine Calculation Decreased Anesthesia-Related Mortality in Mice

**DOI:** 10.3389/fvets.2021.651202

**Published:** 2021-07-22

**Authors:** Carlos Poblete Jara, Rodrigo S. Carraro, Ariane Zanesco, Beatriz Andrade, Karina Moreira, Guilherme Nogueira, Bruno L. Souza, Thais Paulino Prado, Valeria Póvoa, William Velander, Licio A. Velloso, Eliana P. Araújo

**Affiliations:** ^1^Nursing School, University of Campinas, Campinas, Brazil; ^2^Laboratory of Cell Signaling, Obesity and Comorbidities Research Center, University of Campinas, Campinas, Brazil; ^3^Faculty of Medical Sciences, University of Campinas, Campinas, Brazil; ^4^Laboratory of Digital Signal Processing in Real Time, School of Electrical and Computer Engineering, University of Campinas, Campinas, Brazil; ^5^Department of Chemical and Biomolecular Engineering, University of Nebraska, Lincoln, NE, United States

**Keywords:** xylazine, anesthesia, mortality and morbidity, smartphone, mobile app

## Abstract

Currently, experimental animals are widely used in biological and medical research. However, the scientific community has raised several bioethical concerns, such as the number of animals required to achieve reproducible and statistically relevant results. These concerns involve aspects related to pain, discomfort, and unwanted animal loss. Retrospectively, we compare two different approaches for anesthesia dosage: a mobile app for dose calculation and a standard dose calculation. A total of 939 C57BL/6J and Swiss mice were analyzed. We collected data on intraoperative and anesthesia-related mortality as described in electronic or physical handwritten records. Our results showed that the mobile app approach significantly reduces anesthetic-related deaths upon using doses of ketamine and xylazine. The results suggest that anesthesia-related mortality can be minimized even more using information technology approaches, helping to solve an old but transversal challenge for researchers working with experimental mice. The mobile app is a free and open code which could be implemented worldwide as an essential requirement for all anesthetic procedures in mice using xylazine and ketamine combination. As an open code app, the Labinsane initiative could also represent the starting point to unify and validate other anesthetic procedures in different species and strains.

## Introduction

Annually, millions of animals are used for experimental purposes (1–3). Experimental mice are usually anesthetized intraperitoneally with a ketamine and xylazine solution (4–7). An intraperitoneal procedure permits rapid application and fast anesthetic effect. However, the reasons why mice die during the anesthetic procedure could be attributed to the inappropriate ketamine and xylazine dosage, the lack of supplemental oxygen, and attention to details such as body temperature, stress associated with handling, and incorrect intraperitoneal technique. Moreover, previous intraperitoneal ketamine and xylazine combinations have caused several challenges related to a low margin of safety, prolonged recovery, and persistence of lost reflexes in mice (5, 8, 9). The ketamine and xylazine doses range from 60 to 200 mg kg^−1^ of ketamine and 4 to 26.4 mg kg^−1^ of xylazine (4, 5, 8–11). However, serious inconsistent rates of anesthesia-related mortality (0–100%) have been reported (5, 8–11).

Recent studies have shown that the MIT App Inventor, a free and open-source software, can improve the performance and quality of the data analyzed, helping operators to make well-informed decisions (12). A previous report validated a smartphone app for the calculation of CO_2_ in inhalational anesthesia (13), supporting the concept that a mobile app could help operators to calculate individual anesthetic doses. Here, our objective was to develop a mobile app to improve the accuracy of intraperitoneal anesthetic doses for mice. In this way, we used a range of previously tested safe doses (5, 11), herein ketamine (mg kg^−1^), and xylazine (mg kg^−1^), in the following proportions: 70/7, 80/8, and 100/10, which are now contained in the “Labinsane” mobile app. Then, we compared the Labinsane anesthetic protocol to the protocol of 133 mg kg^−1^ ketamine with 27 mg kg^−1^ xylazine.

## Materials and Methods

**Figure 1:**
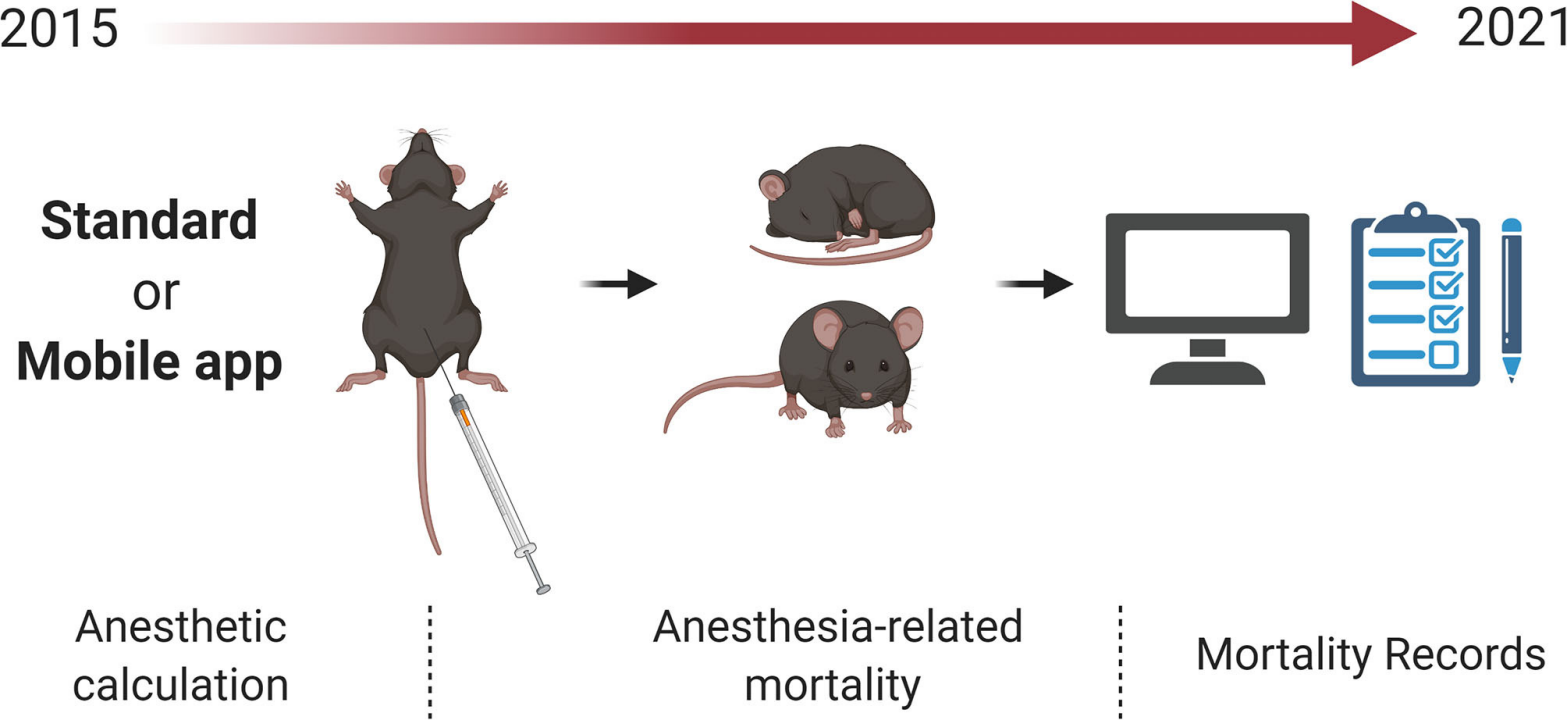
Anesthetic records from C57BL/6J and Swiss mice Collected records from Standard or Mobile app approach of anesthesia-related mortality. Records were collected between 2015 and 2021.

Retrospectively, anesthetic procedure records from C57BL/6J and Swiss mice were included. We collected electronic and physical handwritten records written between 2015 and 2021 from eight researchers (Figure 1). The records included from different projects were approved by the ethical committee of the University Campinas (#4330-1A, #5521-1, #5425-1, #5349-1, #4637-1, #4072-1, #3826-1, #5414-1, #4930-1 and #4699-1). Records of anesthetic procedures were conducted according to the “Guide for the Care and Use of Laboratory Animals” (14). The mice were maintained under specific pathogen-free conditions in individual cages in a regimen of 12-h dark and 12-h light cycles and room temperature of 21°C. All mice had *ad libitum* access to food (3.7 kcal g^−1^) and water.

### Criteria for Inclusion

Anesthetic records from intraoperative mortality were collected. Anesthesia-related mortality was defined as lost breath or rigor mortis up to 2 h after the induction of anesthesia. The anesthetic procedure characteristics were as follows: intraperitoneal route, using an insulin syringe, 31G needle, sterile saline solution or distilled water for injections, 10 g/100 ml stock ketamine and 2 g/100 ml stock xylazine (C57BL/6J), or 10 g/100 ml stock ketamine, 2 g/100 ml stock xylazine, and 0.5 g/100 ml stock diazepam (Swiss). The animals were on a chow diet, and individual weights were measured on the same day of the anesthetic procedures. We collected records from animals of two procedures: two symmetrical full-thickness excisional wounds (6-mm biopsy punch) created on the back of each mouse as described previously (15) and stereotaxic surgery carried out using a stereotaxic frame (16). All surgeries were conducted up to 1 h after anesthesia induction. Immediately after surgery, the mice were placed on a new germ-free surface in a temperature-controlled room (25°C), without oxygen supplementation or monitoring of the temperature of the animal. Furthermore, only male mice were included.

The anesthetic injections are a routine procedure in our lab. Records with the following pattern were selected: using only a mixture of xylazine (Anasedan, Brazil), ketamine (Dopalen, Brazil), with or without diazepam, and saline (0.9%) solution. Intraperitoneal injections were performed in mice in the dorsal recumbent position. The anesthetic combination was made up as a single injection.

### Criteria for Exclusion

All incomplete records were excluded from the analysis: anesthesia-related mortality, lost breath, or rigor mortis occurring after 2 h after induction of anesthesia, mice fed with a high-fat diet, or animals treated with different specific care support before, during, or after the anesthetic procedure such as oxygen supplementation.

### Anesthetic Procedure Screened

**Figure 2:**
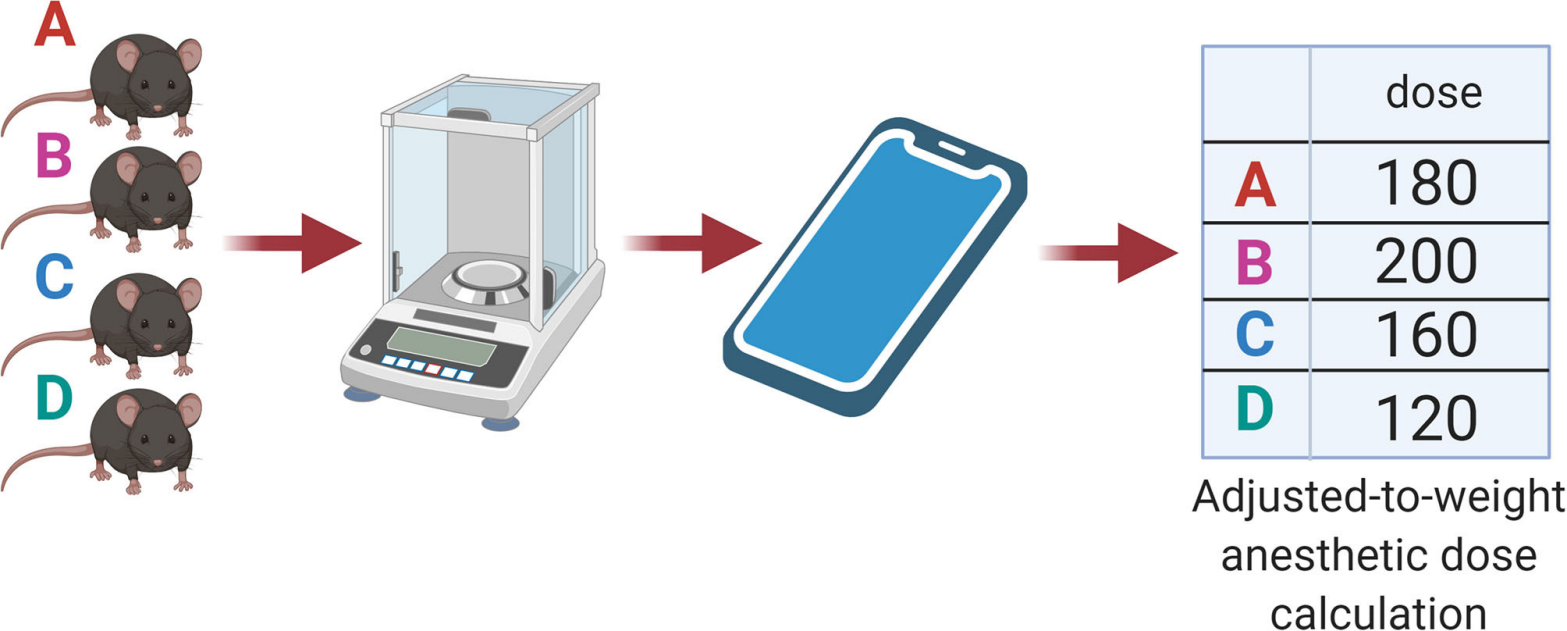
Labinsane schematic workflow. Individual animal weights were processed by the Labinsane mobile app. A master anesthetic cocktail was calculated based on the total number of animals and weights. The Mobile app approach showed an adjusted-to-weight anesthetic dose for each animal.

For both the standard dose and individual dose, the animals were weighed on the same day of the anesthetic procedure. The anesthetic combination was freshly prepared for each experiment, as previously described (10). The final solution was used immediately. Standard doses were prepared as previously described (6), with some local adaptations. Briefly, the final solution of the standard dose was prepared with 400 μl xylazine (2 g/100 ml), 400 μl ketamine (10 g/100 ml), and 200 μl saline solution. The standard dose combination (80–100 μl of the mixture, according to weight) was administered intraperitoneally once by procedure. The final solution of the individual dose was prepared and calculated by the Labinsane mobile app. Briefly, the mobile app processes body weight, calculates a master anesthetic combination, and then indicates the individual volume (in μl) to administer for each individual mouse (Figure 2). A specific individual dose adjusted to the body weight of the mouse was administered intraperitoneally once by procedure. For the C57BL/6 strain, a range of previously tested safe doses was used. Herein, ketamine (mg kg^−1^) and xylazine (mg kg^−1^) were in the following proportions: 70/7, 80/8, and 100/10 (3, 7), while for the Swiss strain, we added diazepam (5 mg kg^−1^) (12).

### Statistical Analyses

Data extracted from the original records were pooled on a Microsoft Excel spreadsheet (Microsoft Corporation, 2007). All statistical tests were performed in GraphPad Prism (6.0v). Data were analyzed with Fisher's exact test (two-sided) and odds ratios. Statistical significance was set as alpha <0.05 and 95% confidence interval (CI). For the linear model, we used a modified Poisson regression model, with robust variance (17). The model was adjusted considering *death* as the dependent variable. The variables “app used”, “strain,” and “age” were also considered as independent variables. In the results, the estimates obtained from the prevalence ratio were presented as well as their respective confidence intervals and *p*-values.

### Labinsane Formula

$$\begin{array}{l} x=A+B+C\\ y=A+B\;+C+D \end{array}$$

The x formula describes the final master anesthetic solution for the C57BL/6 mice. The y formula describes the final master anesthetic solution for the Swiss mice. In both formulas, A represents the final ketamine stock volume, B the final xylazine stock volume, C the final saline volume, and D the final diazepam stock volume.

$$\begin{array}{l} \text{A }=\left(\underset{\text{weigh}}{\sum }\times \frac{\text{KP}}{1,000}\times \text{KC}\right) \end{array}$$

“A” from the Labinsane formula is the sum of all body weights of mice times ketamine prescription times ketamine stock concentration.

$$\begin{array}{l} \text{B }=\left(\underset{\text{weigh}}{\sum }\times \frac{\text{XP}}{1,000}\times \text{XC}\right) \end{array}$$

“B” from the Labinsane formula is the sum of all body weights of mice times xylazine prescription times xylazine stock concentration.

$$\begin{array}{l} \text{D }=\left(\underset{\text{weigh}}{\sum }\times \frac{\text{DP}}{1,000}\times \text{DC}\right) \end{array}$$

“D” from the Labinsane formula is the sum of all body weights of mice times diazepam prescription times diazepam stock concentration.

$$\begin{array}{l} C\;=\left(A+B\right)\times \;4\\ C=\left(A+B+D\right)\times \;3 \end{array}$$

“C” from the Labinsane formula is the sum of the final ketamine stock volume and final xylazine stock volume times the dilution factor. The dilution factor here is a constant: “4” for C57BL/6 mice and “3” for Swiss mice.

The records collected described three different dose prescriptions of ketamine (mg kg^−1^) and xylazine (mg kg^−1^) in the following proportions: 70/7, 80/8, and 100/10, with the same prescription for diazepam (5 mg kg^−1^). The anesthetic dose prescription was defined according to the need for long or short procedures. For the standard protocol, we collected records describing 133 mg kg^−1^ ketamine and 27 mg kg^−1^ xylazine of dose prescription.

## Results

### Labinsane Mobile App Decreased Anesthesia-Related Mortality

Over the 6 years of the study, 939 anesthetized mice were evaluated: 754 C57BL/6 and 185 Swiss mice (Table 1). This study identified 25 intraoperative and anesthesia-related deaths within all anesthetic procedures. The individual protocol showed an anesthesia-related mortality rate of 1.03%. On the other hand, mice anesthetized using the standard protocol showed an anesthesia-related mortality rate of 10.24% (Table 2). The association between the mobile app and survival outcome was statistically significant (Table 2). Moreover, the mobile app approach suggests a protective effect (Table 3), with a prevalence ration of 0.06-fold the anesthesia-related deaths compared to the standard dose procedure (odds ratio, 0.06; 95% CI, 0.02–0.13; *p* < 0.0001). We excluded 123 electronic records of mice fed with a high-fat diet or of animals that received different specific care support such as oxygen supplementation.

**Table 1:** Characteristics of mice.

	**Number of animals**	**Standard**	**Individual**
Average age (weeks ± *SD*)	Swiss (185)	8.0 (0)	8.0 (0)
	C57BL/6J (754)	7.6 (0.5)	8.0 (0)
Sex (%)	Females (0)	0	0
	Males (939)	100	100
Strain (%)	Swiss (185)	49	51
	C57BL/6J (754)	16	84
Average weight (g)	Swiss (185)	33.8	33.5
	C57BL/6J (754)	25.2	25.1
Surgical procedure (%)	Stereotaxic surgery (387)	32.2	67.8
	Full-thickness biopsy (552)	17.2	82.8

*Age, sex, strain, weight, and procedure profile of mice with total number, separated by standard or individual protocol. Values expressed in weeks with standard deviation (weeks ± SD), percentage (%), and grams (g)*.

**Table 2:** Multivariables.

**Variable**	**Death**				***p*-Value**
	**No**		**Yes**		
	***n***	**%**	***n***	**%**	
App used					<0.0001^a^
No	149	89.76	17	10.24	
Yes	765	98.97	8	1.03	
Strain					0.8014^a^
C57	733	97.21	21	2.79	
Swiss	181	97.84	4	2.16	
Concentration					<0.0001^a^
70–100	765	98.97	8	1.03	
133	149	89.76	17	10.24	
Age, weeks					0.0880^a^
7	61	93.85	4	6.15	
8	853	97.60	21	2.40	
Procedure					0.5840^b^
Full-thickness excisional biopsy	535	97.10	16	2.90	
Stereotaxic surgery	379	97.68	9	2.32	

*Descriptions of the variables app used, strain, drug concentration, age, and procedure were presented*.

a *Fisher's exact test*.

b *Chi-square test*.

**Table 3:** Poisson regression.

**Dependent variable**	**Independent variables**	**Prevalence ratio^a^**	**95% CI**		***p*-value**
			**Lower**	**Upper**	
Death^a^	App used (ref = no)	0.06	0.02	0.13	<0.0001
	Strain (ref = Swiss)	2.13	0.39	11.55	0.3797
	Age, weeks (ref = 7)	2.03	0.31	13.18	0.4585
	Procedure (ref = wound)	0.54	0.11	2.61	0.4436

a *The probability of presenting the result “yes” was estimated; n = 939*.

Different doses of ketamine/xylazine for the mobile app protocol (70/7, 80/8, and 100/10 mg/mg per kg) or standard protocol (133/27 mg/mg per kg) were evaluated. Identified as the higher anesthesia-related mortality dose was 133 mg of ketamine/27 mg of xylazine per kilogram when compared to the other weight-adjusted doses (Table 2).

Next, it was evaluated whether different strains, C57BL/6 or Swiss, age, or surgical procedure could influence the anesthesia-related mortality rate. No significant difference in anesthesia-related mortality was identified between C57BL/6 and Swiss mice, 7- and 8-week-old, or between full-thickness excisional biopsy and stereotaxic surgery (Tables 2, 3).

### Labinsane Mobile App Matched All Anesthetic Dose Calculations

To validate the mobile app anesthetic dose calculation, the Labinsane app was challenged to reproduce the results of the individual doses calculated by a Microsoft Excel formula. The Microsoft Excel formula was chosen because it is one of the top stable software available. Randomly, 449 mice were tested, showing that the Labinsane mobile app matched all (100%) the individual anesthetic doses calculated by the Microsoft Excel formula (Table 4) with no statistical difference (*p* = 0.9). These results suggested no errors in the Labinsane mobile app code.

**Table 4:** Labinsane validation.

	**Excel formula**	**Labinsane**
Animals	449	449
Minimum (μl)	114.6	114.6
Median (μl)	130.7	130.7
Maximum (μl)	249.8	250
Mean (μl)	146.1	145.9
Standard deviation (μl)	31.9	31.6
Standard error of the mean (μl)	1.5	1.5
Lower 95% confidence interval (CI; μl)	143	143
Upper 95% CI (μl)	149	148.8

*Number of animals: n, 449. Median, mean, standard deviation, standard error, and confidence interval comparing the Microsoft Excel spreadsheet and Labinsane mobile app. Unpaired t-test: p-value, 0.9432*.

## Discussion

The Principles of Humane Experimental Technique published in 1959 by Russell and Burch proposed that all efforts should be made to minimize the use and suffering of experimental animals in biological and health research (3R). Today, after 60 years, we are still struggling to achieve the high standards idealized by Russell and Burch.

Despite the recommended anesthetic doses being well known worldwide, the final injected doses could be different than those calculated. Several animals for experiments, preparation of master anesthetic solutions, small drug volumes, and the volumetric limitations of syringe systems could interfere in the proper application of the recommended dose rates. We asked whether we could improve the reported average mortality using a ketamine and xylazine combination. First, it was tested in a Microsoft Excel spreadsheet containing the proper prescription formula. However, after implementation, adherence to the new MS Excel-based dose calculation brought new difficulties for operators—for instance, animal biosafety limitations, aseptic concerns about personal computers in laboratories performing animal procedures, low volumes of anesthetic to remove from the flask, highly concentrated residual anesthetic volumes in the syringe, and limitations of syringe systems without the capacity to fractionate small volumes of anesthetics. To solve some of these complexities, we tried a different approach and developed a mobile app. Using the Labinsane mobile app on personal mobile phones, researchers, and operators were able to use personal devices in the workplace. Mobile phones were easily disinfected and speeded up master anesthetic calculations as well as the individual anesthetic dose calculation.

Several advantages of volatile anesthetics over injectables have been shown. However, anesthetic procedures using airways require specific equipment, which can interfere with the experiment result and increases both cost and workspace usage. If volatile anesthetics cannot be applied, injectable anesthesia is indicated (18). Intraperitoneal anesthetic protocols based on the injection of ketamine–xylazine solution are widely used in experiments with rodents due to the low cost, minimum training, and the fact that no equipment is being required (4). Nevertheless, there is a wide variation in the recommended dose, which is possibly due to differences between mouse strains, type, and duration of the procedure, health conditions, age, and research goals (4, 8, 10, 19). Previous reports compared the efficacy of the intraperitoneal and subcutaneous administration of ketamine (100 mg/ml) and xylazine (20 mg/ml) solution for inducing surgical anesthesia. Among C57BL/6, BALB, and ICR mice, no death occurred in the subcutaneous administration groups, while 16.7% (10 of 60 mice) of mice injected intraperitoneally died (10). Specifically, more females died after the intraperitoneal injection compared with males (10). Previous reports have also shown that the ketamine, xylazine, and diazepam combination is effective to induce anesthesia for surgical procedures (20–24).

The principal causes of unsafe medication in humans are related to failures in drug preparation and lack of treatment standardization (25). Experimental animal safety should also ensure the proper anesthetic dose calculation and administration. In this way, the Labinsane mobile app seeks to ensure the safety of C57BL/6 and Swiss mice by pursuing proper individual anesthetic dose calculation and administration.

We decided to use “4” and “3” as dilution factors in the mobile app formulas, with the aim to increase the total volume to administer. By increasing the dilution factor, we decreased the anesthetic agent concentrations in every microliter of the final cocktail. In this way, if any operator mistake occurs, the volume of the anesthetic agents is low (in μl), protecting the mice from overdoses. These dilution factors helped the operators with anesthetic volumes that were easier to fit in common insulin syringes (1 ml). This altogether protects the mice from human mistakes. This approach suggests that the mobile app could improve mice safety related to anesthetic administration. We believe that this method could be better than just calculating the drug dosages on a milligram-per-kilogram basis with standard protocol because it is ready to use, already tested, and could be an open initiative to automate the drug calculation process in other species and strains.

The MIT App Inventor platform (appinventor.mit.edu) allows researchers to easily create new mobile apps. Indeed App Inventor apps have been shown to improve data analysis and help in making well-informed decisions (12). The use of the App Inventor has also contributed to children's learning (26), home automation (27), and self-care actions (28). Our study established that Labinsane, a MIT-based mobile app, helps researchers to markedly reduce anesthesia-related mortality. We encourage researchers to validate new experimental animal strains and species based on modular collaboration with Labinsane to decrease anesthetic-related death.

Due to the retrospective nature of this study, we recommend limiting the interpretation of our results to the variants herein described as well as avoiding extrapolation to other scenarios. The mice in the mobile app protocol received a lower ketamine/xylazine drug concentration compared to the standard protocol. We also collected records only from male mice, and previous reports (10) showed that female mice mortality is higher than male mortality. In this way, future studies will be needed to determine the mobile app accuracy for female anesthesia induction.

The xylazine and ketamine dose regime proposed by the mobile app protocol is lower than the standard dose used, and even the ratio between the dose of xylazine and ketamine between groups is different. In fact, the mobile app protocol proposes a reduction, especially of the xylazine dose. Similar to previous studies, the mobile app protocol introduced a different dose regimen in accordance with the duration of the procedure (4, 8, 10, 19). The xylazine dosage used in the standard protocol was also 0.6 mg/kg higher than the higher dose reported in the literature. In this way, future studies will be necessary to determine the relationship of anesthesia-related mortality with a similar ketamine/xylazine concentration using this mobile app initiative.

## Conclusions

The current study was conducted to validate a mobile app for anesthetic dose calculation. The results of the study demonstrated that a MIT App Inventor-based app decreased anesthesia-related mortality and matched all anesthetic dose calculations. The results suggest that anesthesia-related mortality can be minimized even more using information technology approaches. The open code of Labinsane will be available after publication on the GitHub site (https://github.com/blinkeado/labinsane) for future protocol validation.

## Data Availability Statement

The raw data supporting the conclusions of this article will be made available by the authors, without undue reservation.

## Author Contributions

CJ contributed to conceptualization, formal analysis, methodology, investigation, writing of the original draft, and review and editing of the manuscript. RC contributed to the methodology, investigation, and writing of the original draft. AZ, BA, KM, GN, TP, and VP contributed to the investigation and writing of the original draft. BS contributed to conceptualization and writing of the original draft. WV contributed to review and editing of the manuscript and formal analysis. LV contributed to formal analysis, resources, review and editing of the manuscript, and supervision. EA contributed to conceptualization, methodology, formal analysis, resources, writing of the original draft, review and editing of the manuscript, and supervision. All authors contributed to the article and approved the submitted version.

## Conflict of Interest

The authors declare that the research was conducted in the absence of any commercial or financial relationships that could be construed as a potential conflict of interest.
